# Frameworks for evaluating health research capacity strengthening: a qualitative study

**DOI:** 10.1186/1478-4505-11-46

**Published:** 2013-12-14

**Authors:** Alan Boyd, Donald C Cole, Dan-Bi Cho, Garry Aslanyan, Imelda Bates

**Affiliations:** 1 Manchester Business School, University of Manchester, Booth Street West, Manchester M15 6PB, UK; 2 Dalla Lana School of Public Health, University of Toronto, Health Sciences Building, 402-155 College Street, Toronto, ON M5T 3M7, Canada; 3 Special Programme for Research and Training in Tropical Diseases – TDR, World Health Organization, 20 Avenue Appia, CH-1211, Geneva 27, Switzerland; 4 Department of International Public Health, Liverpool School of Tropical Medicine, Pembroke Place, Liverpool L3 5QA, UK

**Keywords:** Capacity strengthening, Evaluation, Frameworks, Health research

## Abstract

**Background:**

Health research capacity strengthening (RCS) projects are often complex and hard to evaluate. In order to inform health RCS evaluation efforts, we aimed to describe and compare key characteristics of existing health RCS evaluation frameworks: their process of development, purpose, target users, structure, content and coverage of important evaluation issues. A secondary objective was to explore what use had been made of the ESSENCE framework, which attempts to address one such issue: harmonising the evaluation requirements of different funders.

**Methods:**

We identified and analysed health RCS evaluation frameworks published by seven funding agencies between 2004 and 2012, using a mixed methods approach involving structured qualitative analyses of documents, a stakeholder survey and consultations with key contacts in health RCS funding agencies.

**Results:**

The frameworks were intended for use predominantly by the organisations themselves, and most were oriented primarily towards funders’ internal organisational performance requirements. The frameworks made limited reference to theories that specifically concern RCS. Generic devices, such as logical frameworks, were typically used to document activities, outputs and outcomes, but with little emphasis on exploring underlying assumptions or contextual constraints. Usage of the ESSENCE framework appeared limited.

**Conclusions:**

We believe that there is scope for improving frameworks through the incorporation of more accessible information about how to do evaluation in practice; greater involvement of stakeholders, following evaluation capacity building principles; greater emphasis on explaining underlying rationales of frameworks; and structuring frameworks so that they separate generic and project-specific aspects of health RCS evaluation. The third and fourth of these improvements might assist harmonisation.

## Background

Health research capacity strengthening (RCS) is recognised as an important area for action to improve health in low- and middle-income countries and to address global health challenges [1]. Health RCS is, however, a complex and context-sensitive process, requiring a combination of different approaches directed at individual, institutional, and societal levels [2]. Hence, evaluation frameworks can also be very heterogeneous [3].

We understand evaluation frameworks to be documents providing a structure or guidance for those involved in health RCS (e.g., funders, the implementers who are funded to do the RCS, and evaluators of RCS efforts) to evaluate what is being done [4]. Such evaluation frameworks have been suggested as important contributors to the sustainability of an organisation’s evaluation practices, particularly if they meet accepted quality standards and provide comprehensible operational guidelines for staff to follow [5]. Frameworks can clarify which evaluation methods to use for particular purposes and circumstances [5]. Frameworks also have the potential to facilitate sharing and learning [6] within and between the organisations involved, by clearly communicating key aspects of the approach to evaluation. Such clarity can also reduce administrative burdens by informing the rationalisation of data collected from health RCS implementers who receive funding from more than one funder agency [7].

Through the Paris Declaration [8] and the Accra Agenda for Action [9], a large number of countries and international organisations committed to the principle of “harmonisation” in order to increase the effectiveness of international aid and align it with the needs of developing countries. Health RCS funders have subsequently made efforts to coordinate their activities, primarily through the ESSENCE on Health Research initiative [10]. Drawing on member experience with the challenges of evaluating health RCS, ESSENCE has produced a framework for planning, monitoring, and evaluation (PM&E) [11] and encourages all health RCS funders to use it. We collaborated with ESSENCE to explore the use of PM&E frameworks and approaches to evaluating health RCS, and to inform refinements to the ESSENCE PM&E framework.

Unable to find peer reviewed literature describing the role and use of funder evaluation frameworks in evaluations of health RCS, we sought to identify ways in which existing frameworks might be developed in order to better guide RCS planning, monitoring and evaluation; facilitate sharing and learning; and enhance coordination and harmonisation of evaluation across different funding agencies. Our primary objective was to describe and compare key characteristics of health RCS evaluation frameworks: their process of development, purpose, target users, structure, content and coverage of important evaluation issues. Our secondary objective was to conduct a preliminary exploration of the potential and actual use of frameworks to improve planning, monitoring and evaluation practice, focusing particularly on the ESSENCE framework’s attempt to harmonise the evaluation requirements of different funders.

## Methods

We followed a mixed methods approach, using stakeholder engagement to inform and illuminate a formal document analysis. The research was given ethical approval by the University of Toronto Health Sciences Research Ethics Board (reference number 26837).

Health RCS funders, implementers and evaluators were identified through a snowballing process, starting with key contacts from ESSENCE member agencies. Participants were engaged via telephone discussions, meetings at the Global Forum for Health Research 2012 [12], and an online survey. The discussions covered topics such as what frameworks for monitoring and evaluating health RCS participants were aware of, how they used health RCS frameworks, and how the usefulness of those frameworks could be enhanced. The online survey focused mainly on use of the ESSENCE PM&E framework, but also included a question asking what other frameworks respondents used in evaluating health RCS. The survey questions are listed in Additional file 1.

We identified framework documents through the engagement process, both directly and indirectly, by looking for references to frameworks in health RCS evaluation reports commissioned by funders. We selected those documents written in English, which described frameworks meeting our broad definition (see Background section above) and focused mainly on evaluation or monitoring. Nine framework documents [11,13-19] from seven organisations were obtained; all but one are publicly available on a website (Table 1). Other organisations whose websites we searched included the Department for International Development, the Swedish International Development Cooperation Agency, and the Council on Health Research for Development, where we found various documents addressing different aspects of evaluation, but no overarching document attempting to draw them together into a comprehensive framework.

**Table 1:** Frameworks included in the analysis

**Organisation**	**Document title (date)**	**Length (approx. no. of words)**	**Specificity of questions and indicators**	**Matrix/logframe structure**
Ministry of Foreign Affairs of Denmark – Danida	Danida Evaluation Guidelines (2012) [13]	Medium (14,000)	Generic	Intervention logic (input, output, outcome, impact) to inform evaluation design
	Danish Development Cooperation in a Results Perspective: Danida’s Framework for Managing for Development Results 2011–2014 (2011) [14]	Medium (9,000)	Generic	Logical framework/results chain forms conceptual basis
ESSENCE on Health Research	Planning, Monitoring and Evaluation Framework for Capacity Strengthening in Health Research (2011) [11]	Short (4,000)	Health RCS-specific	Matrix with example indicators for activities, outputs and outcomes
The Special Programme for Research and Training in Tropical Diseases co-sponsored by UNICEF, UNDP, World Bank, and WHO (TDR)	Monitor, evaluate, improve: TDR Performance Assessment Framework – Measuring results (2011) [15]	Medium (13,000)	Health research-specific plus health RCS-specific	Matrix with example indicators based on expected results chain
National Institutes of Health: Fogarty International Center (FIC-NIH)	Framework for Program Assessment (Evaluation and Review) (2005) [16]	Short (5,000)	Health research-specific plus training aspect of health RCS	Categories with example indicators
Netherlands Organisation for Scientific Research: WOTRO Science for Global Development	Mid Term Review (2005–2008) form: Testable goals (review questions) (2005)	Very short (2,000)	Health RCS-specific	Indicators for institutional capacity
International Development Research Centre (IDRC)	Framework for evaluating capacity development in IDRC (2005) [17]	Long (24,000)	Capacity strengthening-specific	Conceptual model for the intervention
	The Corporate Assessment Framework (2004) [18]	Very short (2,000)	Implies capacity strengthening-specific plus research-specific	None
Canadian International Development Agency (CIDA)	CIDA Evaluation Guide: Overcoming challenges; Delivering results; Meeting expectations; Making a contribution (2004) [19]	Very long (36,000)	Generic	A logical model should inform data collection for outputs, outcomes and impacts

We analysed the framework documents using a structured qualitative approach [20]. First, we identified potential characteristics of frameworks, issues of concern in health RCS and its evaluation that frameworks might address, and good practices that frameworks might suggest evaluators to adopt. This was done via the stakeholder engagement process, a thematic analysis of publicly available documents produced by funder evaluation departments, such as policies and good practice reports, and an analysis of evaluation frameworks, guides and tools identified from a systematic search of peer reviewed literature [21]. The documents analysed are listed in Additional file 2. Data extraction templates were then developed for general characteristics relating to the framework as a whole, such as its purpose and intended use (Additional file 3), and for specific evaluation issues of concern to funders, the most prominent of which were participation of stakeholders, measurement of impact, opportunities for learning, appropriate timing of the evaluation, technical quality, and equity. Additional file 4 provides descriptions of each of these issues. The good practices associated with each issue were used to guide our assessments of the frameworks. Additional file 5 lists over 50 such good practices, associated with 15 issues. A matrix analysis of within-case and cross-case comparisons [22] was then conducted in order to identify patterns in how the frameworks covered the issues.

## Results

### Purpose and intended users of frameworks

Most (6/9) documents specified the purpose of the framework, including improvement of harmonisation (i.e., synergy, sharing knowledge, and labour) among funders, the promotion of systematic assessment of the funder’s contribution to health RCS, and gaining of a better understanding of the funder’s internal project management and evaluation processes (Table 2). For some frameworks [14,17-19] the purpose was clear from the title (e.g., “Framework for Managing for Development Results”, “Corporate Assessment Framework”). The intended users of the frameworks explicitly stated in six of the framework documents, were primarily funder’s own staff or members of the funders’ consortium (n = 5) or development evaluators (n = 1), though several recognised that others such as project partners, researchers and policy makers may also be interested in using the framework. The majority of framework documents related to either on-going monitoring or one-off, retrospective summative evaluations, usually conducted at the end of the project or programme by external consultants or by the funder’s own evaluation unit.

**Table 2:** Purpose of frameworks and their intended users

**Document**	**Purposes**	**Intended users**
Danida (2012) [13]	“Constitutes the basic framework for evaluations of Danish development cooperation” (p. 3)	“Those who have a professional engagement in evaluation of development cooperation, as well as others interested in evaluation. These include those who are parties to an evaluation process and the users of evaluations. Moreover, the guidelines may be of interest to a broader audience, such as students, researchers and policy makers, and the interested public” (p. 3)
	“Do not constitute a manual in evaluation methods and techniques” (p. 3)	
Danida (2011) [14]	“Know[ing] more about results management and the … approach Danida uses” (p. 1)	“The main audience for the framework is Danida staff” (p. 1)
	“Clarify[ing] how Danida manages the process [of achieving and demonstrating results] towards this goal [of securing value for money and aid effectiveness]” (p. 1)	
ESSENCE (2011) [11]	“To improve harmonization among funders of health research capacity strengthening. Its use should make it easier for recipients of funding to fulfil the PM&E obligations of different funders and facilitate synergy, division of labour and sharing of knowledge among funders” (p. 4)	“[hopefully] ESSENCE members [typically funders] and other partners will have access” (p. 2)
TDR (2011) [15]	“A tool … [that] promotes and guides systematic assessment of TDR’s strategic and technical relevance and contribution towards its vision” (p. 5)	“For use both by TDR staff and the broad range of stakeholders involved in the governance and implementation of TDR’s Ten Year Vision and Strategy” (p. 5)
	“Guides TDR staff and stakeholders through a more systematic way of monitoring and evaluating the Programme’s performance” (p. 6)	
FIC-NIH (2005) [16]	Not explicitly stated. Describes roles and responsibilities in relation to organisational systems and suggests evaluation questions and indicators	Not explicitly stated. Program Officers, Principal Investigators, external evaluators and staff of partner institutions are among those whose roles in assessment are described
WOTRO (2005)	Not explicitly stated. Specifies data to be collected and presented in reviews	Not explicitly stated. The review committee [external evaluators] and programme partners are mentioned in the document
IDRC (2005) [17]	“A generic guide for the assessment of any capacity development activity or project component supported by [IDRC]; and for any form of assessment (formative or summative; monitoring or evaluation)” (p. 2)	Not explicitly stated. Implicitly, anyone assessing any capacity development activity supported by IDRC. Refers to “the evaluator” at points
IDRC (2004) [18]	“Promote coherence between the aims and objectives expressed at the corporate level and those expressed at the program level” (p. 4)	Managers within IDRC. Also briefly mentions roles for program teams, centre support units, and the Board of Governors
	“Help managers make decisions that support programming efforts to achieve the IDRC mission” (p. 2)	
	“Provides a structure for organizing and reporting on results at the corporate level” (p. 2)	
CIDA (2004) [19]	“Ensure that the Agency’s staff, consultants and partners are properly informed about how evaluations of CIDA’s investments … are to be carried out, and what they are expected to achieve” (Foreword)	“Staff, consultants and partners” (Foreword)
	“A thorough reading offers an in–depth understanding of the Agency’s evaluation activities. Or, individual items of interest can be quickly accessed. Uninitiated readers can learn about the fundamentals of the evaluation process, while seasoned practitioners can benefit from normative guidance to complete the task–at–hand” (p. 1)	

### Structure of frameworks

The frameworks tended to specify particular goals that the funding agency wanted to see achieved, together with corresponding indicators, against which evaluations were expected to assess progress. Frameworks varied in the extent to which they considered their own underlying assumptions about evaluation and health RCS, and the need for evaluations to take account of contextual constraints in assessing health RCS projects. One [17] used an explicit conceptual model of the capacity strengthening process to underpin the framework and guide the design of evaluations, drawing attention to aspects such as different learning modalities (informal, non-formal, and formal academic) and four specific management capacities. Some [11,14,15] contained matrix structures similar to logical frameworks (or “logframes”) [23], with columns corresponding to indicators and sources of evidence, but not to assumptions. The intention was for the spaces in the matrix to be filled in for every health RCS programme, project, and activity, with these sometimes nested hierarchically. Others [13,17,19] used a logic model or ‘results chain’ covering input, output, outcome and impact, or similar variants. One had a very practical emphasis, using checklists that helped to explain the organisation’s expectations and to reduce scope for misunderstandings [13].

In some cases, there was a single document devoted to describing the framework, often focusing on evaluation questions, related indicators, and organisational systems for data collection [11,15-16; WOTRO (2005) – Unpublished data]. In others, a relatively brief document or section, which similarly described “results based management” type aspects of the framework, was supplemented by a second document or additional sections providing guidance about evaluation more broadly [13,14,17-19]. Overall, monitoring rather than planning and evaluation, was emphasized in most of the frameworks, though two [15,19] gave roughly equal emphasis to all three components. Additional documents to support planning were seldom signposted in the framework documents, and in most cases we judged that the relationships between planning, monitoring and evaluation had not been made clear.

### Development of frameworks

Five of the frameworks contained some information describing how the framework had been developed (Table 3). Three of these frameworks were produced solely by specialist evaluators, whether internal to the organisation or externally commissioned [13,14,17-19], while development of the other two involved wider consultation, that included funding recipients [11,15].

**Table 3:** Framework development and proposed review processes

**Document**	**Development process**	**Evaluation publications referenced**	**Review process**
Danida (2012) [13]	Produced by the Foreign Ministry’s evaluation unit	Draws heavily on the OECD/DAC quality standards for development evaluation (2010), from which key statements are incorporated	“The guidelines will be updated as need arises, and comments and suggestions for improvements or clarifications are welcome”
	Aspects may have been inspired by participation in peer reviews of other evaluation functions conducted by OECD/DAC and United Nations networks	Refers to its’ own study on conducting evaluations jointly with partner countries	May also learn from the Multilateral Organisations’ Performance Assessment Network (MOPAN) and the multilateral development banks’ Common Performance Assessment System (COMPAS)
		Refers to a small number of academic publications	This 2012 document is a revised version of a document published in 2006
		Signposts material produced by various international development related networks and World Bank initiatives	
Danida (2011) [14]	Not stated	Uses the OECD standard Managing for Development Results (MfDR) as its management strategy	Requests feedback from staff and external partners. Plans to review the performance measurement tools listed
			This 2011 document replaces a document published in 2005
ESSENCE (2011) [11]	“Consultation, first between various ESSENCE members and secondly with a broader group of stakeholders (including African recipients of funding for health research)”	Five publications: one academic article; two reports related to other health RCS funder evaluation frameworks [TDR and IDRC]; two reports by independent policy/practice organisations	“The matrix is planned to be revised periodically. Funders are invited to adopt a learning attitude towards capacity strengthening and to contribute to the continuous improvement of the matrix, based on their own experiences with capacity strengthening Initiatives”
TDR (2011) [15]	Developed by internal working groups, consulting with internal and external stakeholders and advised by an external advisory group. External input was mainly from research institutions, research funding institutions, and development agencies	Fifteen “related documents” are listed. These were produced by other development-related organisations: OECD/DAC, various United Nations programmes and the World Bank	“This framework will need to be continuously reviewed and refined in order to address the Programme needs”
FIC-NIH (2005) [16]	Not stated	None	Not stated. This 2005 document is a revised version of an initial document published in 2002
WOTRO (2005)	Not stated	None	Not stated
IDRC (2005) [17]	Produced by two university-based international development consultants whose expertise included evaluation and monitoring	References a report on outcome mapping published by IDRC	Not stated
	Based on a file review of capacity development in 40 IDRC projects		
IDRC (2004) [18]	Developed by the Senior Management Committee and the Evaluation Unit	None	“CAF is an experiment … and will require refinement on an ongoing basis”
			“The evaluation unit, policy and planning group, and senior management committee will periodically assess the utility of the CAF performance areas, and decide how to make appropriate modifications”
CIDA (2004) [19]	Prepared by the evaluation unit and an external consultant.	References documents drawn from government and other agencies in its own country, and OECD/DAC work	“We welcome any comments and/or suggestions that you may have” [email address provided]

Four frameworks [11,13-15,19] referenced work from outside of the organisation, and three of these [13-15,19] made at least some use of the OECD/DAC quality standards for development evaluation [24]. Reports from other funding agencies and networks were cited much more often than academic research (see Additional file 6, which contains a list of all 35 evaluation resources cited in the framework documents). One of the framework documents [17] was based on formal research into the agency’s monitoring and evaluation practices. While review processes were seldom described in any detail, three documents were revisions or replacements of previous versions [13,14,16].

### Content of health RCS evaluation frameworks

Framework documents tended to be descriptive rather than explanatory. They varied in length from less than 5,000 words to 36,000 words. The “purpose, aims and objectives of the framework”, “quantitative indicators, measures and targets”, and “intended use of the framework” were the best developed, while the “use of theory”, “capacity building to commission or conduct evaluations” and “role allocation and governance” were less well developed. With the exception of measuring impact using quantitative indicators, coverage of aspects of health RCS evaluations that funders valued (stakeholder participation, opportunities for learning, demonstrating equity, quality assurance, and optimising the timing of evaluations) was often limited. Some of these aspects had been incorporated into the frameworks, although equity tended to be limited to an analysis of “south” and “north” representation rather than more holistic application of the concept of equity, to include considerations such as socio-economic strata within a developing country [25].

### Usage of frameworks to improve health RCS planning, monitoring, and evaluation

The frameworks generally focused on the specific systems and processes of the particular funder organisation. Three documents contained some information about how to use the framework in practice and three further documents referenced sources of information relevant to aspects of evaluation practice (Additional file 6 lists all sources of information referenced by the frameworks). One [17] explained underlying rationales for using the framework and another [19] provided helpful and detailed information to support the conduct of evaluations. Consideration of the ability of stakeholders to contribute to the evaluation process was minimal, although some organisations did recognise that capacity building of partners and their systems for conducting or participating in evaluations might be needed [13,19], and some documents provided glossaries, diagrams and checklists to aid understanding and use (Table 4, column 4).

**Table 4:** Characteristics of individual frameworks related to harmonisation and to building evaluation capacity

**Document**	**Coordination and alignment**	**Capacity building to commission or conduct evaluations**	**“How to do it” information provided to support Framework use or PM&E practice**
Danida (2012) [13]	Whole chapter on multilateral development coordination. Highlights benefits of using country systems and data, and of joint or coordinated PM&E	Mentions the need to assess team capacity for qualitative evaluation and the cultural competence of data collectors. Mentions that it may develop the capacity of country organisations it works with on evaluations	Five annexes cover key issues with regard to codes of conduct; quality control and assurance; project inception reporting; evaluation reporting; analytical quality
Danida (2011) [14]	Some material about coordinating multilateral projects. Highlights benefits of using partners’ monitoring systems	Mentions the possible need to develop capacity for output monitoring among partners	Provides links to tools that funder staff may use, particularly for monitoring
ESSENCE (2011) [11]	Emphasises need for harmonization of practices across different funders and them using the framework in partnership	Paragraph on general capacity strengthening for funders, but nothing specific to evaluation	Little practical detail. Some key concepts regarding indicators are clarified. There is a list of sources, but this does not indicate which provide practical guidance
TDR (2011) [15]	Mentions need for partnership across funders	Not mentioned	Contains quite detailed instructions, plus a clear and fairly comprehensive glossary. There is a reading list, but this is not prominent and does not indicate which documents provide practical guidance
FIC-NIH (2005) [16]	Emphasises stakeholder involvement in planning only	Training and support for funder staff is provided by the Evaluation Officer; support for other stakeholders not mentioned	Little detail. Provides most on indicators, giving examples, but not how to identify and construct an indicator
WOTRO (2005)	Not mentioned	Not mentioned	No information to support practice
IDRC (2005) [17]	Not mentioned	Mentions that health RCS may need to address monitoring capacity	Explains the thinking behind CS evaluation, relationships between PM&E, and the types of questions to ask, providing examples of particular questions
IDRC (2004) [18]	Not mentioned	Not mentioned	Provides a link to characteristics of good performance and associated monitoring questions. Nothing apart from this
CIDA (2004) [19]	Not mentioned	It takes the form of a capacity building tool. Some discussion about building capacity among local recipients	The entire document focuses on providing detailed information to support the conduct of CIDA evaluations. There are checklists for each chapter, and a list of acronyms

Several of the evaluation reports which we analysed were commissioned by organisations whose frameworks we also analysed (Danida [13,14], IDRC [17,18], NIH-FIH [16], WOTRO [unpublished data], and TDR-WHO [15]). For all except WOTRO [unpublished data], however, the evaluations began before the studied versions of the frameworks were published. Few explicit mentions of specific funder evaluation frameworks were made, although some referred to “frameworks” generally as a way of facilitating systematic data collection and thereby improving evaluation quality.

In the 15 months between its’ publication and the date of our survey, the ESSENCE PM&E framework [11] had been used by four out of the twenty responding organisations. Two had used it as a central organising framework for their evaluation activities. Some organisations had been unaware of the framework, perhaps because their evaluations had been conducted prior to its publication. However, the most frequent reason given for not using the ESSENCE framework was that a different framework was already being used (8 of the 12 respondents who gave a reason). Comments suggested that wider use of the ESSENCE framework was limited by the circumstances of individual funders; for example, when RCS was not exclusively focused on health, when another framework was already in active use, or when tailoring might be required.

“*We would need to customise the ESSENCE Framework to … allow us the flexibility of incorporating some of our grant conditions into [our] monitoring and evaluation activities.*” (Policy maker)

Three-quarters of the funding organizations (15/20) agreed that more supporting guidance, tools or training, and greater emphasis on learning and qualitative aspects of evaluation would make wider, or more in-depth, usage more likely.

“*Officers … need to have guidance on effective use of [the ESSENCE framework]. It will help too if this framework [an updated version] were built on existing ones that officers are used to. People are hesitant to change old ways but would try if they see familiar zones… This is important especially if it has to be self-tutored.*” (University implementer).

### Diversity and strengths of frameworks

The PM&E frameworks we analysed had different strengths (Table 5). For example, the Canadian International Development Agency’s (CIDA) framework [19] provided comprehensive and detailed information and checklists to support use of the framework in practice and addressed issues of quality and validity. Efforts to harmonise frameworks between organisations were more prominent in the newer frameworks (Table 4, column 2). Many of the framework documents referred to reports produced by other funders or to funder evaluation networks. The development of the Special Programme for Research and Training in Tropical Diseases (TDR)s framework [15] involved some other funders. Only the ESSENCE framework [11], however, had been specifically produced as a collaborative effort among funders.

**Table 5:** Relative strengths of frameworks

**Document**	**Strengths**
Danida (2012) [13]	References/links to further information, e.g., on coordination and alignment
	Structured plan for reviewing/developing the framework
	Explicit use of OECD/DAC quality standards
	Addresses quality and validity
Danida (2011) [14]	No particular strengths identified
ESSENCE (2011) [11]	Short
	Some emphasis on planning
	Indicators are health RCS-specific; includes examples
	Stakeholder involvement in developing the framework
TDR (2011) [15]	Some health RCS-specific indicators; includes examples
	Accessibility – glossary, diagrams
	Stakeholder involvement in developing the framework
	Some consideration of the impact of the funding agency’s own systems
FIC-NIH (2005) [16]	Short
	Some consideration of the impact of the funding agency’s own systems
WOTRO (2005)	Short
	Indicators are health RCS-specific
IDRC (2005) [17]	Capacity-strengthening specific indicators
	Based on consideration of the specific processes of capacity-strengthening, equivalent to a conceptual model.
	Based on in depth research of the agency’s experiences
	Provides detailed information to support practice
IDRC (2004) [18]	Short
CIDA (2004) [19]	Emphasis on planning
	Emphasis on building evaluation capacity
	Accessibility/checklists
	Provides detailed information to support practice
	Addresses stakeholder participation issues
	Addresses equity issues, including gender
	Guidance on data collection and quantitative measures/indicators
	Some guidance on qualitative data
	Guidance on making comparisons and judgements
	Addresses quality and validity
	Some use of theory
	Guidance on learning
	Guidance on timing and timescales

## Discussion

### Improving health RCS evaluation frameworks

Despite most of the health RCS evaluation framework documents studied being available to the public, and sometimes being used by evaluators and research capacity strengtheners, they were predominantly intended to fulfil the needs of the funder agency, with an emphasis on gathering data to monitor achievement of corporate goals. Most of the documents did not provide in-depth guidance about how to implement the frameworks in practice, thereby potentially limiting the extent to which stakeholders beyond the funding agencies, such as funding beneficiaries, could participate constructively in the evaluation process. Such participation can facilitate ownership of the evaluation, thereby promoting learning, implementation of recommendations, and sustainable change [26]. Better use of diagrams, glossaries, checklists, and links/references to further information, could promote more systematic implementation of the frameworks.

The provision of such additional information combined with training in evaluation, would be a particularly important consideration for funding organisations keen to encourage participation of stakeholders in the evaluation process. Greater stakeholder involvement in evaluation and framework development, to include disadvantaged or marginalised groups, could also help frameworks to address equity issues more fully. Data from various sources, including framework documents themselves, funder policy documents, external evaluation reports and contacts in funder agencies, indicated a need to build evaluation capacity within all organisations involved in health RCS. Funding agencies might benefit from explicitly instituting a strategy of evaluation capacity strengthening to underpin their framework development, though this might require changes to organisational cultures, structures and practices [27]. Ideally, frameworks for planning, monitoring, and evaluating health RCS efforts should be easily accessible to stakeholders and facilitate high quality data collection and analysis, which may necessitate different documents for different purposes and audiences.

There was, however, also substantial diversity among the frameworks, and our categorisation of this diversity provides an opportunity for funders to compare frameworks and potentially identify improvements (using Table 5, for example). Another way forward might be for funders to place more emphasis on explaining the rationales underlying their frameworks and their provenance. Making explicit the underlying assumptions and logic models can facilitate understanding, learning, and development, and help to identify appropriate indicators [28,29]. Doing so could also contribute to the development of evaluative thinking within and across funding agencies and health RCS implementers.

Despite differences in funders’ organisational cultures and ways of working with frameworks, the telephone discussions and meetings with stakeholders conducted as part of this research demonstrated a desire among funding organisations for health RCS evaluations to be productive for their own organisation, and collectively through harmonisation efforts. Some funders had revised their framework documents, and in addition to the ESSENCE on Health Research initiative members’ efforts to harmonise their PM&E frameworks, there were other examples of research funders [13-15] using common resources to inform monitoring and evaluation activities, such as the OECD/DAC standards [24]. This demonstration of potential transferability of methods and tools for health RCS PM&E suggests that there may be opportunities for more formal sharing of resources and frameworks between funding organisations and for inter-organisational learning, which might be facilitated by the ESSENCE initiative.

Although the ESSENCE PM&E framework [11] was developed jointly by several funding agencies, it had not been widely used in practice, predominantly because funders were already using alternative frameworks which better matched their history or needs. Building on the fact that some frameworks are already informed by common resources, a pragmatic and useful approach may be to have a two-part framework consisting of generic issues, which may be transferable between projects or even funders, and project-specific issues, which are unique to each context and health RCS initiative. Since this research was conducted, some ESSENCE members have adapted parts of the framework for use with projects they fund. ESSENCE members are also considering using the results of the research to inform a review of their policies and practices.

### Study weaknesses and strengths

We did not study some unpublished frameworks, and may have overlooked some not written in English. We may also have missed some other potentially relevant documents (e.g., internal reports reviewing framework use, describing developments, or communicating frameworks to funder staff, funded organisations or contracted evaluators), and documents not oriented primarily to evaluation. Our data on the use of frameworks derived from evaluation reports mostly relates to large, formal evaluations conducted by external consultants, who may not have chosen to work with existing frameworks. There may be greater use of frameworks in smaller scale evaluations conducted by funder staff.

On the other hand, we used multiple data sources from a variety of funders of health RCS evaluations, including consultations, a survey, framework documents, and evaluation reports. It is unlikely that we have missed any English language health RCS evaluation frameworks that are commonly used beyond a single funding agency. Frameworks not analysed may therefore be less likely to have an external focus and to be accessible to stakeholders.

### Future directions

Little is known about the roles that frameworks play in establishing identities, roles, values, practices, and relationships with regard to monitoring and evaluation, evaluation capacity building, and “harmonisation” (i.e., normalisation of a particular set of evaluation values and practices), and how they are used by specialist evaluators, funder organisation staff, and non-specialist evaluators in funded organisations in order to design and conduct evaluations. This is an important knowledge gap that could be addressed by collecting more in-depth information using ethnographic approaches and qualitative methods, by analysing a wider range of documents, including internal reports, policies and plans, training materials and documents not written in English, and by extending our online survey to consider frameworks other than the ESSENCE framework.

We believe that action research into how organisations develop their health RCS frameworks, and the benefits and constraints of the different types of frameworks, would also be beneficial. This knowledge would help organisations to develop frameworks that are underpinned by an explicit rationale and which acknowledge any underlying assumptions, thus facilitating more informed and appropriate use. The potential for health RCS evaluation frameworks to strengthen evaluation capacity, to improve the evaluation culture within organisations, and to facilitate sharing of funders’ approaches to health RCS evaluations, needs to be exploited so that meaningful evaluation findings can be generated jointly by health RCS funders and the organisations that they fund.

Our research focused on evaluation frameworks for health RCS due to our particular interest in this topic and the health remit of the research funder. While there are some aspects of health RCS, such as infrastructure for clinical trials and associated ethical issues, that are different to RCS more widely, there is also much that is common, and indeed most of the frameworks we studied were generic. Future research might usefully consider a wider range of RCS frameworks.

## Conclusions

This article breaks new ground by describing the key characteristics of funder evaluation frameworks, and how they are used to support evaluation of health RCS. We have identified potential avenues for further research on evaluation frameworks, and aspects of frameworks that might be usefully developed. Overall, we believe that there is scope for improving frameworks through the incorporation of more accessible information about how to do evaluation in practice; greater involvement of stakeholders, following evaluation capacity building principles; greater emphasis on explaining underlying rationales of frameworks; and structuring frameworks so that they separate generic and project-specific aspects of health RCS evaluation. The third and fourth of these improvements might assist harmonisation.

## Abbreviations

CIDA: Canadian International Development Agency; Danida: Ministry of Foreign Affairs of Denmark; ESSENCE: ESSENCE on Health Research initiative; FIC-NIH: National Institutes of Health: Fogarty International Center; IDRC: International Development Research Centre; PM&E: Planning, monitoring and evaluation; RCS: Research capacity strengthening; TDR: The special programme for research and training in tropical diseases; WOTRO: Netherlands Organisation for Scientific Research: WOTRO Science for Global Development.

## Competing interests

Garry Aslanyan is part of the secretariat of the ESSENCE on Health Research initiative and an employee of TDR-WHO.

## Authors’ contributions

The research was conceived and designed by AB, DCC, GA, and IB. The data was analysed and synthesised by AB, DCC, DBC, and IB. The manuscript was drafted by AB, DCC, and IB. All authors were involved in data collection, revising the manuscript, and reading and approving the final manuscript.

## Supplementary Material

Additional file 1 Online survey questions. Click here for file

Additional file 2 References used to inform framework characteristics, PM&E issues and associated good practices. Click here for file

Additional file 3 Data collected to describe framework characteristics. Click here for file

Additional file 4 Health RCS evaluation issues of concern to funders. Click here for file

Additional file 5 Data collected to assess how frameworks address specific PM&E issues and associated good practices. Click here for file

Additional file 6 Evaluation resources cited in the framework documents. Click here for file
