# Supplementary material for: Frameworks for evaluating health research capacity strengthening: a qualitative study
Source: Health Res Policy Syst. 2013 Dec 14;11:46. doi: 10.1186/1478-4505-11-46 (PMC3878679; doi:10.1186/1478-4505-11-46)
Supplement: Additional file 1 — Online survey questions. [file 1478-4505-11-46-S1.pdf]

Default Question Block

What is the name of your organization?

Block 2

During the last year, has ${q://QID3/ChoiceTextEntryValue} engaged in any of the following activities?

	Yes	Unsure	No
HRCS planning	◯	◯	◯
HRCS monitoring	◯	◯	◯
HRCS evaluation	◯	◯	◯

Block 3

Did ${q://QID3/ChoiceTextEntryValue} draw upon or use the ESSENCE PM&E Framework as part of its HRCS planning activities?

Yes	Unsure	No
◯	◯	◯

Did ${q://QID3/ChoiceTextEntryValue} draw upon or use the ESSENCE PM&E Framework as part of its HRCS monitoring activities?

Yes	Unsure	No
◯	◯	◯

Did ${q://QID3/ChoiceTextEntryValue} draw upon or use the ESSENCE PM&E Framework as part of its HRCS evaluation activities?

Yes	Unsure	No
◯	◯	◯

Block 4

The ESSENCE PM&E Framework has 3 parts:

1. **Principles: participation and alignment; understanding the context; building on strengths; long-term commitment; interlinked capacity components; continuous learning; harmonization**
2. **Indicator matrix: for activities, outputs and outcomes at individual, organisational and national and regional research system levels**
3. **Lessons learned template**

Which parts of the ESSENCE PM&E Framework did ${q://QID3/ChoiceTextEntryValue} use?

	Yes	Unsure	No
Principles	◯	◯	◯
Indicators	◯	◯	◯
Lessons learned	◯	◯	◯

Block 5

Please describe how the ESSENCE PM&E principles were used by ${q://QID3/ChoiceTextEntryValue}, covering the following points:

- **The situation**
- **The purpose of using the principles**
- **Who used the principles**
- **How they used the principles**
- **What the results of using the principles were**

How helpful were the ESSENCE PM&E principles?

Very helpful	Quite helpful	Neither helpful nor unhelpful	Quite unhelpful	Very unhelpful
○	○	○	○	○

What advice would you give to others who might be thinking of using the ESSENCE PM&E principles?

Please describe how the ESSENCE PM&E indicators were used by ${q://QID3/ChoiceTextEntryValue}, covering the following points:

- **The situation**
- **The purpose of using the indicators**
- **Who used the indicators**
- **How they used the indicators**
- **What the results of using the indicators were**

How helpful were the ESSENCE PM&E indicators?

Very helpful	Quite helpful	Neither helpful nor unhelpful	Quite unhelpful	Very unhelpful
○	○	○	○	○

What advice would you give to others who might be thinking of using the ESSENCE PM&E indicators?

Please describe how the ESSENCE PM&E lessons learned template was used by ${q://QID3/ChoiceTextEntryValue}, covering

the following points:

- **The situation**
- **The purpose of using the template**
- **Who used the template**
- **How they used the template**
- **What the results of using the template were**

How helpful was the ESSENCE PM&E lessons learned template?

Very helpful	Quite helpful	Neither helpful nor unhelpful	Quite unhelpful	Very unhelpful
○	○	○	○	○

What advice would you give to others who might be thinking of using the ESSENCE PM&E lessons learned template?

Block 6

Why did ${q://QID3/ChoiceTextEntryValue} not draw upon or use the ESSENCE PM&E Framework?

	Yes	Unsure	No
Did not know about it	○	○	○
Not applicable to our programs	○	○	○
We use a different Framework	○	○	○
Other reasons (please describe below)	○	○	○

Please describe ${q://QID3/ChoiceTextEntryValue}'s reasons for not using the ESSENCE PM&E Framework

Please describe briefly the other frameworks that ${q://QID3/ChoiceTextEntryValue} uses to support PM&E

What would make ${q://QID3/ChoiceTextEntryValue} more likely to use the ESSENCE PM&E Framework?

	Yes	Unsure	No
More publicity about the Framework	○	○	○
Modifications to the Framework	○	○	○
More supporting guidance, tools or training	○	○	○
Other things (please describe below)	○	○	○

Please explain the reasons for your answers to the previous question

Block 1

Would you like to receive a copy of the final report of our research?

- Yes
- No

Contact details

- Name
- Job title
- Email
