# Supplementary material for: Frameworks for evaluating health research capacity strengthening: a qualitative study
Source: Health Res Policy Syst. 2013 Dec 14;11:46. doi: 10.1186/1478-4505-11-46 (PMC3878679; doi:10.1186/1478-4505-11-46)
Supplement: Additional file 2 — References used to inform framework characteristics, PM&E issues and associated good practices. [file 1478-4505-11-46-S2.docx]

**References used to inform framework characteristics, PM&E issues and associated good practices**

Adrien MH. (2003) *Evaluation Capacity Development: Guide to conducting reviews of organizations supplying M&E training.* Washington: The World Bank; 2003.

Afsana K, Habte D, Hatfield J, Murphy J, Neufeld V. *Partnership Assessment Toolkit.* Ottawa: Canadian Coalition for Global Health Research; 2009. Available from: <http://members.ccghr.ca/Resources/Documents/Resources/PAT_Interactive_e.pdf> [accessed 2013 Apr 26]/

Bates I, Akoto AY, Ansong D, Karikari P, Bedu-Addo G, Critchley J, Agbenyega T, Nsiah-Asare A. (2006) Evaluating health research capacity building: an evidence-based tool. *PLoS Med* 2006;3:e299. doi:10.1371/journal.pmed.0030299

Bates I, Ansong D, Bedu-Addo G, Agbenyega T, Akoto AY, Nsiah-Asare A, Karikari P. Evaluation of a learner-designed course for teaching health research skills in Ghana. *BMC Med Educ* 2007;7(18): n.p. doi:10.1186/1472-6920-7-18

Centers for Disease Control and Prevention. *Framework for program evaluation in public health[ MMWR 148 (No. RR-11)].* Atlanta: CDC; 1999. Available from: <http://www.cdc.gov/mmwr/preview/mmwrhtml/rr4811a1.htm> [accessed 2013 Apr 26].

Cohen AM, Adams B. Process Evaluation: Rationale and Application. In Love A, editor. *Evaluation Methods Sourcebook (Vol. 1).* Ottawa: Canadian Evaluation Society; 1991. pp.44-67.

Conrad KJ, Randolph FL, Kirby MW, Bebout RR. Creating and using logic models: Four perspectives. *Alcsm Treat Quart* 1999;17:17-31.

Cooke J. A framework to evaluate research capacity building in health care. *BMC Fam Pract* 2005:6(44). doi:[10.1186/1471-2296-6-44](http://dx.doi.org/10.1186%2F1471-2296-6-44)

Davies R, Dart J. *The 'Most Significant Change' (MSC) Technique: A Guide to Its Use.* Published Online; 2005. Available from: <http://www.mande.co.uk/docs/MSCGuide.pdf> [accessed 2013 Apr 26].

Horton D, Alexaki A, Bennett-Lartey S, Brice KN, Campilan D, Cardon F, et al. Using and Benefiting from Evaluation. In Horton D, Alexaki A, Bennett-Lartey S, Brice KN, Campilan D, Cardon F, et al. *Evaluating capacity development: Experiences from research and development organizations around the world*. Ottawa: International Development Research Centre; 2003. pp.107-19. Available from: http:⁄⁄www.idrc.ca/en/ev-31556-201-1-DO_TOPIC.html [accessed 2013 Apr 26].

Horton D. *Planning, Implementing and evaluating capacity development.* The Hague: International Service for National Agricultural Research; 2002. Available from: <http://www.seachangecop.org/sites/default/files/documents/2002%2007%20Planning%2C%20Implementing%2C%20and%20Evaluating%20Capacity%20Development.pdf> [accessed 2013 Apr 26].

OECD/Development Assistance Committee. *Quality Standards for Development Evaluation*. Published online: OECD Publications; 2010. Available from: http://www.oecd.org/dac/evaluation/qualitystandardsfordevelopmentevaluation.htm [accessed 2013 Apr 26].

Panel on Return on Investment in Health Research. *Making an Impact: A Preferred Framework and Indicators to Measure Returns on Investment in Health Research.* Ottawa: Canadian Academy of Health Sciences; 2009. Available from: <http://www.cahs-acss.ca/wp-content/uploads/2011/09/ROI_FullReport.pdf> [accessed 2013 Apr 26].

Saunders M, Houghton AM, Davies P, Morgan L, Pearce L, Marsden R. *Evaluation Capacity Building ECB 'Toolkit'.* Lancaster: Lancaster University; 2010. Available from: <http://www.lancs.ac.uk/fass/events/capacitybuilding/toolkit/> [accessed 2013 Apr 26].
