# Supplementary material for: Frameworks for evaluating health research capacity strengthening: a qualitative study
Source: Health Res Policy Syst. 2013 Dec 14;11:46. doi: 10.1186/1478-4505-11-46 (PMC3878679; doi:10.1186/1478-4505-11-46)
Supplement: Additional file 3 — Data collected to describe framework characteristics. [file 1478-4505-11-46-S3.docx]

**Data collected to describe framework characteristics**

| **Characteristic** | **Data collected** |
| --- | --- |
| Name | 1. Title of the framework document, including any subtitles 2. Explanations for the choice of name |
| Purpose, aims and objectives | 1. Both explicit statements and extracts (E.g. outcomes of using the framework) that suggest other objectives 2. Issues the framework is intended to address 3. What prompted the development of the framework? 4. Which of planning, monitoring and evaluation (PM&E) does the framework cover? Definitions of these terms, and what is written about the relationships between them? 5. Activities other than PM&E that the framework is intended to guide (E.g., “reviews”). Definitions of these other activities, and how they relate to PM&E. 6. How programs and projects are defined and distinguished. How the framework related to programs and projects. |
| Underlying principles or values | 1. Both explicit statements and extracts that imply other values |
| Intended use | 1. How the framework is intended to be used and in what circumstances 2. Who is intended to use the framework (E.g., funder staff, implementers, external evaluators) 3. Assess the usefulness and accessibility of the framework to all stakeholders (cf Afsana et al, 2009)) 4. Draw out any “program theories” underlying the framework 5. Illustrative examples 6. The process for implementing the framework (or the approaches it suggests) across the organisation (E.g., identification of skills, resources and experience needed to use the framework; provision of support such as training; related initiatives to complement the framework) |
| Generality | 1. Is the framework specific to health research capacity strengthening (HRCS), to RCS, or to CS, or is it a general document on PM&E? 2. Is it specific to programs, or to projects? 3. Which aspects of the framework are specific? Which are general? 4. Guidance about adapting the framework (or PM&E) to local circumstances and learning about HRCS (cf Horton, 2002) |
| Development process | 1. The organization that commissioned the framework, including which units or staff did the commissioning 2. The organization that produced the framework, including which units or staff did the work 3. Sources drawn on to inform commissioning or production (E.g. references cited, stakeholders consulted) 4. Support from senior managers (cf Horton, 2002) |
| Revisions | 1. Publication dates of current and previous versions 2. How versions differ and why the changes were made 3. Plans for monitoring, evaluating and revising the framework |
| Information to support PM&E practice | 1. Guidance about how to actually do PM&E. How prescriptive is it? How flexible is it (cf Afsana et al, 2009) 2. Guidance about how to commission evaluations. 3. How checklists, illustrative examples and diagrams are used 4. Signposting of further sources of information, tools, advice or training 5. Help with terminology (e.g., list of abbreviations; glossary; definitions of terms, including PM&E terms) (cf Saunders et al, 2010). Other provision to increase accessibility to stakeholders. 6. Word count of the document |
