# Supplementary material for: Frameworks for evaluating health research capacity strengthening: a qualitative study
Source: Health Res Policy Syst. 2013 Dec 14;11:46. doi: 10.1186/1478-4505-11-46 (PMC3878679; doi:10.1186/1478-4505-11-46)
Supplement: Additional file 4 — Health RCS evaluation issues of concern to funders. [file 1478-4505-11-46-S4.docx]

**HRCS evaluation issues of concern to funders**

| **Issue** | **Description** |
| --- | --- |
| Participation | Stakeholder participation in evaluation has many potential benefits but is likely to require investments of time and resources. It may be difficult to reconcile with keeping evaluation costs low, and with demonstrating accountability through independent, external evaluation. |
| Impact | Evaluation should help to demonstrate value for money. This requires a comprehensive, planned approach to evaluation, which considers short-, medium- and long-term outcomes and collects both quantitative and qualitative data, underpinned by a model or programme theory linking HRCS to outcomes. Resources and capacity to conduct such evaluation are however limited. |
| Learning | Evaluation should produce learning. With ongoing participatory evaluation, HRCS programmes and projects can continuously improve themselves; and evaluation of pilots and model/theory-based evaluation can facilitate wider learning if findings are shared horizontally and vertically, within and between organisations. Current organisational systems and cultures may however not be conducive to such learning. |
| Timescale | Evaluation should be designed at the planning stage as an integrated part of the HRCS programme or project, be conducted regularly throughout its course, and continue long enough for impacts to have had time to be achieved. This requires funder flexibility and long-term commitment. |
| Quality and validity | Evaluation should be both reliable and produce valid conclusions, but these aims can be in tension. Reliability suggests having a small number of quantitative indicators, but qualitative methods are needed to produce valid findings about complex HRCS initiatives. This makes evaluation complex. |
| Equity | Evaluation should address potential inequities in the research and HRCS processes, such as between genders, minority and majority groups, people speaking different languages, and researchers/institutions from the South and North participating in research partnerships. Involving disadvantaged groups in evaluations can help, but in addition to the challenges of participation generally (see above), it may be hard to identify which groups and inequalities are relevant. |
