# Supplementary material for: Frameworks for evaluating health research capacity strengthening: a qualitative study
Source: Health Res Policy Syst. 2013 Dec 14;11:46. doi: 10.1186/1478-4505-11-46 (PMC3878679; doi:10.1186/1478-4505-11-46)
Supplement: Additional file 5 — Data collected to assess how frameworks address specific PM&E issues and associated good practices. [file 1478-4505-11-46-S5.docx]

**Data collected to check how frameworks address specific PM&E issues and associated good practices**

| **Issue** | **Good practices** |
| --- | --- |
| PM&E purpose, aims and objectives | 1. Clarify rationale and purpose. Consider why the evaluation is being conducted now, who will benefit, and how it is to be used for: learning, accountability (cf OECD/DAC, 2010); planning, managing, developing (cf Saunders et al, 2010) 2. State specific objectives in order to clarify what the evaluation aims to find out (cf OECD/DAC, 2010) 3. State and justify the evaluation criteria, explicitly considering relevance, efficiency, effectiveness, impact and sustainability (OECD/DAC, 2010) |
| PM&E of the support that the funding organisation provides | 1. Consider process and outcome evaluation of both projects and the overarching program, including funder systems (cf Cooke, 2005) |
| Capacity building to commission or conduct evaluations | 1. Consider building evaluation capacity among HRCS recipients (cf Horton, 2002), implementers and funders, both prior to, and as a consequence of the evaluation (cf OECD/DAC, 2010) |
| Co-ordination and alignment | 1. Take into account national and local evaluation plans, activities and policies (OECD/DAC, 2010) 2. Take into account PM&E practices of co-funders 3. Consider of joint evaluation across funders and partner countries (OECD/DAC, 2010) |
| Role allocation and governance | 1. Consider who should play what role (e.g., when should external evaluators be commissioned? (Saunders et al, 2010) What skills and experience should evaluators have? How should conflicts of interest be dealt with?) 2. Match the monitoring process to the capacity available (cf Davies and Dart, 2005) 3. Document the purpose and expectations, agreeing clear terms of reference between commissioners and evaluators (OECD/DAC, 2010) 4. Select the evaluation team openly to have a mix of evaluation skills, subject knowledge and local knowledge, giving consideration to gender balance (OECD/DAC, 2010) 5. Consider the independence of evaluators from stakeholders, addressing conflicts of interest openly. Consider how stakeholder cooperation with evaluators will be assured (OECD/DAC, 2010) 6. Consider how a free, open and independent evaluation process will be assured, in order to enhance credibility of the evaluation (OECD/DAC, 2010) 7. Ensure resources (funds, staff and skills) are adequate (OECD/DAC, 2010) 8. Design governance and management structures to fit the evaluation objectives, and safeguard credibility, inclusiveness and transparency (OECD/DAC, 2010) 9. Implement the evaluation efficiently within the allotted time and budget; discussing and agreeing any variation (OECD/DAC, 2010). |
| Participation | 1. Consider the level of involvement in PM&E of stakeholders such as HRCS beneficiaries and disadvantaged communities using, e.g., Arnstein’s ladder, including considering a partnership approach (OECD/DAC, 2010) 2. Provide support/resources/capacity development to enable stakeholder involvement throughout the PM&E process, including broad-based consultation prior to any program (Afsana et al, 2009; OECD/DAC, 2010) 3. Consider the contributions that external partners can make (Horton, 2002) 4. Protect participants’ rights and welfare through formal arrangements (OECD/DAC, 2010; Afsana et al, 2009) 5. Seek stakeholders’ opinions and incorporate their comments into the evaluation report at the draft stage (OECD/DAC, 2010; Adrien, 2003) |
| Equity (including gender) | 1. Identify and address power inequalities 2. Abides by relevant ethical guidelines, respect all stakeholders and make the design sensitive to sociological differences (E.g., gender roles, ethnicity, ability, age, sexual orientation, age) (OECD/DAC, 2010) 3. Present disaggregated data to clarify any differences between sexes and between different groups of poor people, including excluded groups. (OECD/DAC, 2010) 4. Include evaluation questions that address cross-cutting issues, such as gender, environment and human rights (OECD/DAC, 2010) |
| Data collection | 1. Identify what data should be collected, when, by what means etc., and how it should be stored, for what length of time, etc. 2. Use multiple data sources (E.g. for triangulation) |
| Quantitative indicators, measures and targets | 1. Be clear about the role that indicators will play within PM&E, bearing in mind their strengths and limitations. 2. Include both process and outcome indicators (Cooke, 2005) 3. Conceptualise outcomes in terms of client need (Cohen and Adams, 1991) 4. Use already validated outcome measures where possible (Bates et al, 2006), with new indicators meeting accepted criteria, E.g., SMART; “unbiased” and robust (OECD/DAC, 2010) 5. Involve stakeholders in the design of indicators in order to promote motivation and commitment (Horton et al, 2003) |
| Qualitative data | 1. Be clear about the role that qualitative data will play within PM&E, bearing in mind their strengths and limitations. 2. Use qualitative data to identify unexpected changes and provide a rich picture (Davies and Dart, 2005) 3. Capture stories as a source of hypotheses about how things work 4. Complement quantitative indicators in order to provide a sufficient basis for decision making (Centers for Disease Control and Prevention, 1999) |
| Comparisons/judgements | 1. Consider stakeholder values in order to identify appropriate standards (Davies and Dart, 2005; Centers for Disease Control and Prevention, 1999) 2. Use sound methods (E.g. modelling) and for setting expected achievement levels against which success, progress etc will be judged. 3. Use sound methods for analysing evaluation data 4. Use sound methods for identifying other initiatives or models to benchmark against 5. Be analytical rather than collecting data because it is there, etc. (Davies and Dart, 2005) |
| Quality/validity | 1. Make systematic and objective assessments of the worth (OECD/DAC, 2010) 2. Exercise quality control throughout the process (OECD/DAC, 2010) 3. Consider whether an evaluation is a feasible way to answer the questions posed (OECD/DAC, 2010) 4. The evaluation questions (translated from the objectives) inform the approach and methodology (OECD/DAC, 2010) 5. Give consideration to a wide range of approaches, weighing up the costs and benefits, and what will work in the local context 6. Have clear criteria for prioritising evaluations and setting the boundaries of evaluations (Panel on Return on Investment in Health Research, 2009) 7. Use appropriate sampling strategies (purposive or random) (E.g. to avoid bias) |
| Use of theory | 1. Develop and use appropriate logic models that include the context, underlying theory/assumptions, intervention and outcomes (Conrad et al, 1999). Where possible do not start from scratch. 2. Clearly define the intervention, including a description of the intervention logic or theory and discrepancies between planned and actual implementation (OECD/DAC, 2010; Conrad et al, 1999) |
| Learning | 1. Devise mechanisms for sharing and learning from the evaluation, based on assessments of relevance (Saunders et al, 2010) and stakeholder capacity to learn. 2. Provide feedback to stakeholders 3. Disseminate the evaluation systematically and in a timely fashion, both internally and externally (OECD/DAC, 2010) 4. Institute a formal and systematic response to and follow-up on evaluation recommendations (OECD/DAC, 2010) 5. Produce the evaluation report in understandable, appropriate forms (OECD/DAC, 2010) |
| Timing | 1. The timing of evaluation takes account of decision-making cycles and the life cycle of projects (Saunders et al, 2005) |
