# Supplementary material for: Frameworks for evaluating health research capacity strengthening: a qualitative study
Source: Health Res Policy Syst. 2013 Dec 14;11:46. doi: 10.1186/1478-4505-11-46 (PMC3878679; doi:10.1186/1478-4505-11-46)
Supplement: Additional file 6 — Evaluation resources cited in the framework documents. [file 1478-4505-11-46-S6.docx]

**Evaluation resources cited in the framework documents**

Active Learning Network for Accountability and Performance in Humanitarian Action [Internet]. London: ALNAP; c1999- [accessed 2013 Apr 26].

Bamberger M, Rugh J, Mabry L. Chapter Seven: Strengthening the Evaluation Design and the Validity of the Conclusions. In: Bamberger M, Rugh J, Mabry L. *RealWorld Evaluation: Working under Budget, Time, Data and Political Constraints.* California: Sage Publications; 2006. p.132-55.

Canadian Evaluation Society [Internet]. Renfrew: Canadian Evaluation Society; c2001- [cited 2013 Apr 26]. *CES Guidelines for Ethical Conduct*. Available from: <http://www.evaluationcanada.ca/site.cgi?section=5&ssection=4&_lang=an>

Cooke J. A framework to evaluate research capacity building in health care. *BCM Fam Pract*. 2005;6(44). doi: [10.1186/1471-2296-6-44](http://dx.doi.org/10.1186%2F1471-2296-6-44)

Danida (Ministry of Foreign Affairs of Denmark). *Evaluation study on experiences with conducting evaluations jointly with partner countries*. Copenhagen: Danida; 2009 Jun. Available from: <http://www.oecd.org/derec/denmark/43962974.pdf> [accessed 2013 Apr 26].

Earl S, Carden F, Smutylo T. *Outcome Mapping: Building learning and reflection into development programs*. Ottawa: International Development Research Centre; 2001.

Gerring J. What is a case study and what is it good for? *Am Polit Sci Rev* 2004 May;98(2):341-54.

Gerring J. *Case study research: principles and practices*. New York: Cambridge University Press; 2007.

International Initiative for Impact Evaluations [Internet]. Washington: 3iE; c2012- [accessed 2013 Apr 26]. Available from: http://www.3ieimpact.org/en/.

Kusek JZ, Rist RC. *Ten Steps to Results-Based Monitoring and Evaluation System: a Handbook for Development Practitioners*. Washington: The World Bank; 2004.

Managing for Development Results [Internet].No Location: MfDR; c-2002 [accessed 2013 Apr 26]. *Common Performance Assessment System (COMPAS)*. Available from: <http://www.mfdr.org/Compas/index.html>.

Multilateral Organisations’ Performance Assessment Network [Internet]. No Location: MOPAN; c2011- [accessed 2013 Apr 26]. Available from: <http://www.mopanonline.org/home>.

Neilson S, Lusthaus C. *IDRC Supported Capacity Building: Developing a Framework for Capturing Capacity Changes*. Ottawa: Universalia Consultants; 2007. Available from: <http://web.idrc.ca/uploads/user-S/11762347991CB-Developing_Framework_Capturing_Capacity_Changes_FINAL.pdf> [accessed 2013 Apr 26]

OECD/Development Assistance Committee [Internet]. Paris: OECD; c2000- [accessed 2013 Apr 26]. *OECD/DAC Evaluation Network.* Available from: <http://www.oecd.org/dac/evaluationnetwork>.

OECD/Development Assistance Committee [Internet]. Paris: OECD; c2000- [accessed 2013 Apr 26]. *OECD/DAC Evaluation Resource Centre (DEReC).* Available from: [www.oecd.org/dac/evaluationnetwork/derec](http://www.oecd.org/derec/derechomepage.htm).

OECD/Development Assistance Committee. *Managing Joint Evaluations*. Published Online; 2010 Jun. Available from: <http://www.oecd.org/dac/evaluation/dcdndep/46868375.pdf> [accessed 2013 Apr 26]

OECD/Development Assistance Committee. *Working Party on Aid Evaluation - Glossary of Key Terms in Evaluation and Results Based Management.* Paris: OECD; 2002. Available from: <http://www.oecd.org/development/peer-reviews/2754804.pdf> [accessed 2013 Apr 26]

OECD/Development Assistance Committee. *Principles for Evaluation of Development Assistance*. Paris: OECD; 2008 [reprint from 1991]. Available from: http://www.oecd.org/dataoecd/31/12/2755284.pdf [accessed 2013 Apr 26].

OECD/Development Assistance Committee. *Quality Standards for Development Evaluation*. Published online: OECD Publications; 2010. Available from: http://www.oecd.org/dac/evaluation/qualitystandardsfordevelopmentevaluation.htm [accessed 2013 Apr 26].

Ortiz EF, Kuyama, S, Munch W, Tang G. *Implementation of Results-Based Management in the United Nations Organizations: Part I - Series on Managing for Results in the United Nations System*. Geneva: United Nations Joint Inspection Unit; 2004. Available from: ftp://ftp.fao.org/docrep/fao/meeting/009/J4769e/J4769e06.pdf [accessed 2013 Apr 26].

Simister N. *Monitoring and Evaluating Capacity Building: Is it really that difficult?* International NGO Training and Research Centre (INTRAC): Oxford; 2010. Available from: <http://www.intrac.org/data/files/resources/677/Praxis-Paper-23-Monitoring-and-Evaluating-Capacity-Building-is-it-really-that-difficult.pdf> [accessed 2013 Apr 26].

TDR. *Performance Assessment Framework of the Special Programme for Research and Training in Tropical Diseases.* Geneva: World Health Organization; 2010.

UNICEF. Programming Tools [Chapter 6]: Integrated Monitoring and Evaluation Plan [Section 7]. In UNICEF. *Programme Policy and Procedures Manual: Programme Operations*. New York: UNICEF; 2007 [revised from 2000]. pp.184-191. Available from: <http://www.unicef.org/tdad/unicefpppmanualfeb07.pdf> [accessed 2013 Apr 26].

UNICEF. *Understanding Results Based Programme Planning and Management*. Published online: Evaluation Office and Division of Policy Planning; 2003. Available from: http://www.unicef.org/evaluation/files/RBM_Guide_20September2003.pdf [accessed 2013 Apr 26].

UNDP. *Handbook on Planning, Monitoring and Evaluating for Development Results*. New York: UNDP; 2009. Available from: <http://web.undp.org/evaluation/handbook/documents/english/pme-handbook.pdf> [accessed 2013 Apr 26].

UNDP. *RBM in UNDP: Selecting Indicators.* New York: UNDP; 2002. Available from: www.undp.org/eo/documents/methodology/rbm/Indicators-Paperl.doc*‎* [accessed 2013 Apr 26].

United Nations Evaluation Group. *UNEG Ethical Guidelines for Evaluation*. New York: UNEG; 2008. Available from: http://www.unevaluation.org/ethicalguidelines [accessed 2013 Apr 26].

United Nations Evaluation Group. *Norms for Evaluation in the UN System*. New York: UNEG; 2005. Available from: http://www.uneval.org/normsandstandards/index.jsp?doc_cat_source_id=4 [accessed 2013 Apr 26].

United Nations Evaluation Group. *UNEG Principles of Working Together*. New York: UNEG; 2007. http://www.unevaluation.org/papersandpubs/documentdetail.jsp?doc_id=96 [accessed 2013 Apr 26].

United Nations Evaluation Group. *Standards for Evaluation in the UN System*. New York: UNEG; 2005. http://www.uneval.org/normsandstandards/index.jsp?doc_cat_source_id=4 [accessed 2013 Apr 26].

Watson D. *Monitoring of Capacity and Capacity Development: ECDPM Discussion Paper 58B.* Maastrich: European Centre for Development Policy Management (ECDPM); 2006.

The World Bank [Internet]. Washington: The World Bank Group; c2005- [accessed 2013 Apr 26]. *Development Impact Evaluation initiative* (DIME). Available from: [www.worldbank.org/dime](http://www.worldbank.org/dime).

The World Bank [Internet]. Washington: The World Bank Group; c2007- [accessed 2013 Apr 16]. *Spanish Impact Evaluation Fund*. Available from: <http://go.worldbank.org/APDX4VZTG0>.

The World Bank. *Sourcebook on Emerging Good Practices: Emerging Good Practice in Managing For Development Results*. 1^st^ Edition. Washington: The World Bank; 2006. Available from: http://www.mfdr.org/Sourcebook/1stEdition/MfDRSourcebook-Feb-16-2006.pdf [accessed 2013 Apr 26].

The World Bank Independent Evaluation Group. Appendix A: Overview of Monitoring and Evaluation in the World Bank . In World Bank Independent Evaluation Group. *2006 Annual Report on Operations Evaluation.* Washington: The World Bank; 2006. pp. 43-50. Available from: http://lnweb90.worldbank.org/oed/oeddoclib.nsf/DocUNIDViewForJavaSearch/112A9879737627728525733100564D70/$file/aroe_2006.pdf [accessed 2013 Apr 26].
